# A GLOBAL AGETECH AGENDA

**DOI:** 10.1093/geroni/igad104.1601

**Published:** 2023-12-21

**Authors:** Andrew Sixsmith, Sally Fowler-Davis, Sadiq Bhanbhro, Rebecca White

**Affiliations:** Simon Fraser University, Vancouver, British Columbia, Canada; Sheffield Hallam University, Sheffield, England, United Kingdom; Sheffield Hallam University, Sheffield, England, United Kingdom; Simon Fraser University, Vancouver, British Columbia, Canada

## Abstract

AgeTech is increasingly seen as a way to support and enhance the health and independence of older people. However, AgeTech research and innovation agendas overwhelmingly focus on the needs of older people in developed countries and have so far failed to recognise that population aging is a global-level trend that intersects with other “megatrends”, such as climate change, urbanisation and international migration. Drawing on an environmental scan of the current and emerging AgeTech sector, this paper argues that technology research, development and innovation needs a global agenda that engages with initiatives such as the UN’s Strategic Development Goals and Decade for Healthy Ageing. It is also important to situate AgeTech in the wider debate on global disparities. The ongoing impact of colonial legacy (afterlife colonization) continues to reduce life chances, leads to poor health, and is present in everyday relationships between people and institutions at global and local levels. This is particularly apparent in the unequal impact of climate change on people and places, but as yet, the discussion of these issues is notably absent in the narratives surrounding AgeTech. This misses significant opportunities for global reciprocity in health, co-development of useful and appropriate technologies, and more sustainable approaches to innovation (e.g opportunities for frugal innovation), The paper highlights the potential role of older people as active participants in technological change, for example for mitigating climate change and its unequal impact on the Global South

